# Association of genetic variants of GRIN2B with autism

**DOI:** 10.1038/srep08296

**Published:** 2015-02-06

**Authors:** Yongcheng Pan, Jingjing Chen, Hui Guo, Jianjun Ou, Yu Peng, Qiong Liu, Yidong Shen, Lijuan Shi, Yalan Liu, Zhimin Xiong, Tengfei Zhu, Sanchuan Luo, Zhengmao Hu, Jingping Zhao, Kun Xia

**Affiliations:** 1 The State Key Laboratory of Medical Genetics, School of Life Sciences, Central South University, Changsha, Hunan, China; 2 Mental Health Institute, The Second Xiangya Hospital, Central South University, Hunan, China; 3 The Xiangya Hospital, Central South University, Hunan, China; 4 The Third Xiangya Hospital, Central South University, Hunan, China; 5 College of Life Science and Technology, Xinjiang University, Xinjiang, China; 6 Key Laboratory of Medical Information Research (Central South University), Changsha, Hunan, China

## Abstract

Autism (MIM 209850) is a complex neurodevelopmental disorder characterized by social communication impairments and restricted repetitive behaviors. It has a high heritability, although much remains unclear. To evaluate genetic variants of GRIN2B in autism etiology, we performed a system association study of common and rare variants of GRIN2B and autism in cohorts from a Chinese population, involving a total sample of 1,945 subjects. Meta-analysis of a triad family cohort and a case-control cohort identified significant associations of multiple common variants and autism risk (P_min_ = 1.73 × 10^−4^). Significantly, the haplotype involved with the top common variants also showed significant association (P = 1.78 × 10^−6^). Sanger sequencing of 275 probands from a triad cohort identified several variants in coding regions, including four common variants and seven rare variants. Two of the common coding variants were located in the autism-related linkage disequilibrium (LD) block, and both were significantly associated with autism (P < 9 × 10^−3^) using an independent control cohort. Burden analysis and case-only analysis of rare coding variants identified by Sanger sequencing did not find this association. Our study for the first time reveals that common variants and related haplotypes of GRIN2B are associated with autism risk.

Autism (OMIM#209850) is a complex neurodevelopmental disorder, characterized by social and language communication impairments and restricted repetitive patterns of behavior^1^. It appears in early childhood, with a typical onset before the age of 3 years old, and shows a remarkable sex bias, with a male excess estimated at 3–4:1^2^,^3^. The prevalence of autism spectrum disorders has risen to 1 in 68 according to the most recent estimates reported by the United States Centers for Disease Control and Prevention^4^. While it is believed that both genetic and environmental factors contribute to the etiology of autism, a recent study revealed that the narrow-sense heritability of autism is approximately 52.4%, which is mostly attributed to common genetic variants or their interactions with environmental factors^5^. Rare de novo mutations contribute substantially to individual liability, but their contribution to variance in liability is only 2.6%^5^.

De novo loss-of-function mutations have been recurrently identified by exome sequencing at several genes, including GRIN2B. Tarabeux et al. first identified one de novo mutation of GRIN2B in a patient with autism^6^. Subsequently, O'Roak et al. identified three de novo loss-of-function mutations and one de novo missense mutation of GRIN2B using exome and targeted sequencing^7^. The observed number of de novo mutation events was significantly higher at GRIN2B than expected on the basic of the mutation rates estimated for each gene^8^.

GRIN2B encodes an NR2 subunit of N-methyl-d-aspartate receptors (NMDARs), a major class of excitatory glutamate receptors in the central nervous system. NMDARs are thought to be tetramers, assembling as a pair of dimers formed from NR1, NR2 and NR3 subunits. The NR2 subunit (GRIN2A, GRIN2B, GRIN2C, or GRIN2D) is the predominant excitatory neurotransmitter receptor in the mammalian brain, acting as the agonist -binding site for glutamate^9^. Disruption of NMDARs causes abnormal synaptogenesis and an imbalance between excitatory and inhibitory currents, which is important for the pathogenesis of autism^10^,^11^. While de novo rare mutations of GRIN2B have been identified in autism patients, common variants and rare inherited variants have not yet been systematically investigated. In this study, we examined the association of common and rare variants of GRIN2B with autism risk in Han Chinese populations.

We performed an association analysis in two sample cohorts to search for common variants associated with autism. One cohort, consisting of 275 case-parents triad families (n = 825), was analyzed using a transmission disequilibrium test (TDT); the other cohort, consisting of cases and controls (n = 1,120), was analyzed using logistic regression (method). A meta-analysis of the two cohorts was performed using the Stouffer combined method to obtain combined evidence for genetic associations with autism. Sanger sequencing was then conducted on 275 probands from the triad families (methods). Common variant association analysis of the coding variants was performed using an independent control cohort. Burden and case-only analyses were evaluated for the rare variants identified by Sanger sequencing.

## Results

### Common variants and related haplotypes are associated with autism

**Figure 1:**
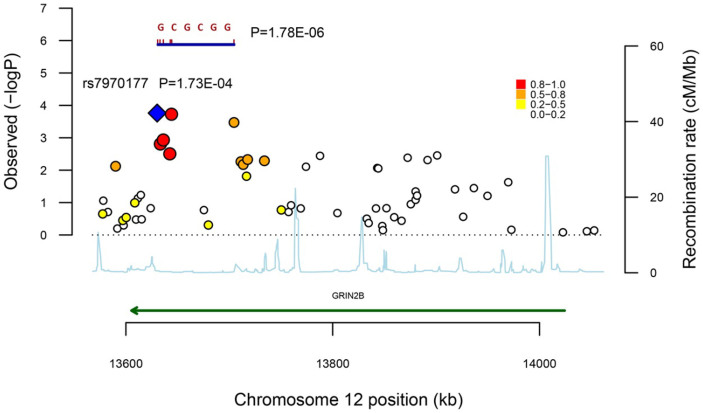
Regional association plot of a negative logarithm of combined P-values for GRIN2B common variants. The most significant SNP was rs7970177 (P = 1.73E-04), which showed strong LD with its nearby five SNPs (r^2^ > 0.8). The six SNPs constructed a strong LD block and showed strong associations with autism (P = 1.78E-06).

**Figure 2:**
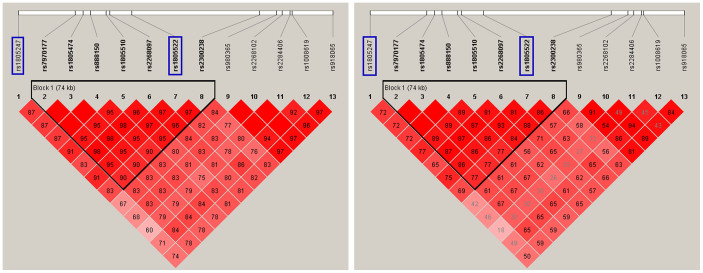
Haplotype plot for the LD block constructed from 11 significant SNPs. SNPs with blue squares were identified by Sanger sequencing and showed significant association. The six SNPs included by the black triangle (Block 1) constructed the most significant haplotype identified by sliding-window analysis.

In total, 74 single-nucleotide polymorphisms (SNPs) were included for the association analysis after strictly quality controls (method) in both case-parents triad family and case-control cohort. All SNPs were located in non-coding regions. TDT analysis of the triad family cohort identified 19 SNPs with nominal significance associations (P.trios < 0.05, Table 1). Logistic regression analysis of the case-control cohort identified seven SNPs showing significant associations (P.cc < 0.05, Table 1). To validate the association results and to reduce the possible false positives, we combined the results of the two cohorts for meta-analysis, and 23 SNPs showed significant associations (P.comb < 0.05, Table 1, Figure 1). Of these, 19 SNPs showed significant associations after correcting for multiple testing (P.adj < 0.05, Table 1). Most of the significantly associated SNPs (n = 11) were located in a LD block (Table 1, Figure 2). Therefore, we performed haplotype association analysis using the sliding-widow method in PLINK, followed by meta-analysis. The most significant haplotype, GCGCGG, was observed at six SNPs in strong LD (rs7970177|rs1805474|rs888150|rs1805510|rs2268097|rs2300238, D′ > 0.9, r^2^ > 0.8, P = 1.78E-06). (Table 2, Figure 1). In addition to the SNPs located in the LD block, there were also five independent association signals (Table 1), including rs7961819 (P = 0.0261), rs2216128|rs2192973 (P = 0.0261 and P = 0.02242, r^2^ = 0.9, D′ = 0.997), rs2300266|rs11055625 (P = 0.02625, r^2^ = 0.993, D′ = 0.997), rs2160732 (P = 0.02242) and rs1861787| rs2284428 (P = 0.02242, r^2^ = 0.870, D′ = 0.979).

Sanger sequencing of the coding and splicing regions in the 275 triad probands identified four common (minor allele frequency [MAF] > 0.05) coding variants, all of which were synonymous (Table 3). To determine whether these coding common variants are associated with autism, we performed association analysis by logistic regression using Asian samples (CHB, CHS and JPT) from the 1000 genome project as controls. Two variants showed significant associations (c.T4197C, rs1805247, MAF = 0.2028, P = 0.0015, odds ratio [OR] = 0.59; c.1806C > T, rs1805522, MAF = 0.1871, P = 0.0042, OR = 0.62; Table 3). The association was still significant after correcting for multiple testing (c.T4197C, P.adj = 0.0061; c.1806C > T, P.adj = 0.0083). These two variants were in strong LD (D′ = 0.91, r^2^ = 0.75) and were located in the autism-related LD block identified above (Figure 2). Both rs1805247 (D′ = 0.87, r^2^ = 0.72) and rs1805522 (D′ = 0.95, r^2^ = 0.86) were in strong LD with the top association signal (rs7970177) of the autism-related LD block. This result further validated the association of this haplotype with autism risk.

### Rare variants of GRIN2B are not associated with autism risk

In addition to common variants identified in the coding regions by Sanger sequencing, we also identified seven rare coding variants (MAF < 0.01), including four synonymous variants and three missense variants (Table 4). Two missense variants (c.A4015G:p.M1339V, c.C3818A:p.T1273K) were not reported (dbSNP138 and ESP6500). Both were inherited from an asymptomatic father. To test whether rare variants of GRIN2B are associated with autism risk, we first performed burden analysis using Asian samples (CHB, CHS and JPT) from the 1000 genome project as controls. Burden analysis identified no significant difference in the burden of rare variants between cases and controls (P = 0.42, Table 4). We then performed a uniq (case-only) analysis to test whether autism patients carried more case-uniq variants. However, no significance was observed (P = 0.47, Table 4).

## Discussion

In this study, both TDT analysis of the triad family cohort and regression analysis of the case-control cohort identified multiple SNPs with significant associations. After further meta-analysis by combining the results from both cohorts and correcting for multiple testing, 19 SNPs showed significant associations. Importantly, 11 SNPs were located in a LD block. The six SNPs with the GCGCGG haplotype were strongly associated with autism.

Sanger sequencing of the coding and splicing regions in the 275 triad probands identified four common variants. Association analysis confirmed two significant associated variants, rs1805247 and rs1805522. Variant rs1805522 was located between the first and second transmembrane segment (M1 and M2, respectively). M1 and M2, combined with a pore helix and pore loop, form the narrowest part of the ion channel pore, which determines the narrow constriction and ion selectivity of the channel^12^. Variant rs1805247 was located at a conserved carboxy-terminal domain (CTD), which has an important role in its interaction with specific signaling proteins, such as CaMKII, SAP102, PSD-95, α-Actinin and Ras-GRF1^13^. These two variants were located in the LD block constructed by 11 significant SNPs. These results further validated the association of the haplotype with autism risk. Interestingly, Yoo et al. reported a five-SNP haplotype association of GRIN2B with autism in Koreans^12^, and their associated haplotype shared the same SNPs rs1805247 and rs1805522 with our results. All evidence indicated that multiple common variants of *GRIN2B* and related haplotypes were associated with autism risk.

Sanger sequencing also identified two missense variants (c.A4015G:p.M1339V, c.C3818A:p.T1273K) that were inherited from an asymptomatic father. These missense variants were also located in the conserved intracellular CTD. It was reported that GRIN2B C-terminally truncated mice die shortly after birth; the lethal phenotype of NR2B C-terminally truncated mice might be caused by impaired intracellular signaling due to the missing intracellular receptor domain^14^. Further investigation is still needed.

Dozens of genome-wide association studies have revealed that coding synonymous variants or common variants lying outside of protein-coding regions are functional^15^. Although we cannot determine the specific biological significance of the significant variants we identified in the current study, they may be located in gene regulation elements; however, this possibility remains unconfirmed. For example, the variants might be involved in the risk of autism by regulating GRIN2B expression. Further study should be conducted to reveal the functional consequences of these variants as related to autism risk.

## Methods

### Subjects

Subjects used for the common variants association study included one cohort of 275 case-parent triad families and one cohort of case-controls (n = 1,120) from the Chinese population. The detailed sample recruitment and diagnosis was described in our previous paper^16^. In summary, all patients were diagnosed with the Diagnostic and Statistical Manual of Mental Disorders-IV criteria (DSM-IV-TR) for autistic disorder by senior psychiatrists from the Psychiatric department of the Second Xiangya Hospital. Patients with fragile X syndrome, tuberous sclerosis, chromosomal abnormality, dysmorphic features, or any other neurological conditions suspected to be associated with autism were excluded. In addition, none of the patients was known (according to the parents' reports) to have any other abnormalities. Subjects used for Sanger resequencing for the coding regions included 275 patients from the 275 triad families in the above common variants association study. All participants provided written informed consent. This study was approved by the institutional review board at the State Key Laboratory of Medical Genetics. All methods were performed in accordance with approved guidelines.

### Genotyping, quality control and Sanger resequencing

All autism cases and controls were genotyped using the Illumina HumanHap CNV370Quad BeadChip or Illumina HumanHap 610Quad BeadChip, as described in our previous paper. Detailed genotyping, quality controls and population stratification analysis were also described. We selected variants within in a 30-kb distance of GRIN2B gene regions. There are 100 variants in the Illumina HumanCNV370Quad BeadChip within this region. After a series of quality controls (SNPs were zeroed out if Mendelian errors >5%, genotype rate >5% and minor allele frequency >0.05), 74 variants remained for association analysis.

For the 275 probands for Sanger resequencing, all exons, flanking splicing sites and untranslated regions (UTRs) of the *GRIN2B* gene (NM_000834.3) were amplified by polymerase chain reaction (PCR). PCR primers were designed using the online Primer3 program (http://frodo.wi.mit.edu/). The PCR products were verified by 6% polyacrylamide gel electrophoresis. Sanger sequencing was performed using an ABI 3100/3130 DNA analyzer. All identified variants were confirmed by repetitive independent PCR amplification and DNA bidirectional sequencing.

### Statistical analysis

Common variant association analysis was performed using PLINK^17^. The TDT was used for the case-parent triad cohort, and logistic regression analyses were used for the case-control cohort. The combined P values from both cohorts were calculated using Stouffer's Z-score method for meta-analysis. The haplotype analysis was performed using up to 10-SNP sliding window approach, followed by meta-analysis of haplotype association results.

Case-control association analyses for the common coding variants identified by Sanger sequencing were performed using logistic regression analysis in PLINK. Rare variants identified by Sanger sequencing were analyzed using PLINK/SEQ (http://atgu.mgh.harvard.edu/plinkseq/index.shtml). Chinese samples from the 1000 genome project (CHB & CHD, n = 286) were selected as controls in the above analysis. The false-discovery rate (FDR) procedure, proposed by Benjamini and Hochberg (1995), was applied for handling multiple comparisons problems.

The regional association plot and haplotype plots were generated using R (http://www.r-project.org/) and Haploview^18^, respectively.

## Author Contributions

Study design: K.X. and H.G. Collecting the samples and performed the experiments: Y.P., J.C., J.O., Y.P., Q.L., Y.S., L.S., Y.L., Z.X., T.Z., S.L., Z.H. and J.Z. Data interpretation and analysis: Y.P., J.C. and H.G. Wrote the manuscript: H.G., Y.P. and J.C. All authors have read and approved the final manuscript.

## Figures and Tables

**Table 1:** Results of single point association analysis for the triad family cohort and the case-control cohort and combined meta-analysis

CHR	SNP	BP	A1	A2	MAF	OR.trios	P.trios	OR.cc	P.cc	P.comb	P.adj
12	rs4763351	13686475	A	G	0.488	1.19	0.1545	1.04	0.7677	0.22411	0.32709
12	rs10845801	13691340	A	G	0.292	1.09	0.5169	1.17	0.2324	0.19266	0.29847
*12*	*rs7961819*	*13698642*	*G*	*A*	*0.201*	*0.65*	*0.00845*	*0.83*	*0.2526*	*0.00755*	*0.0261*
12	rs12814951	13700576	A	C	0.217	1.03	0.8332	1.07	0.6342	0.62741	0.69143
12	rs2160517	13705892	A	G	0.494	0.85	0.1987	1	0.9793	0.35384	0.43426
12	rs2193149	13706502	A	G	0.304	1.09	0.5078	1.04	0.771	0.50024	0.57475
12	rs966664	13709208	A	G	0.485	0.88	0.3	0.95	0.6485	0.29133	0.3933
12	rs1806201	13717508	A	G	0.496	1.21	0.1232	1.1	0.4406	0.10199	0.20397
12	rs1806202	13718561	A	C	0.205	1.1	0.5214	1.11	0.465	0.33203	0.41697
12	rs1806213	13723977	C	A	0.21	1.11	0.4773	1.1	0.5007	0.32775	0.41697
*12*	*rs7970177*	*13738988*	*A*	*G*	*0.192*	*0.54*	*0.00039*	*0.75*	*0.07802*	*0.00017*	*0.005*
*12*	*rs1805474*	*13742150*	*A*	*C*	*0.194*	*0.67*	*0.01395*	*0.71*	*0.04361*	*0.00155*	*0.01673*
*12*	*rs888150*	*13745044*	*A*	*G*	*0.194*	*0.67*	*0.01395*	*0.7*	*0.03264*	*0.00116*	*0.01562*
*12*	*rs1805510*	*13751252*	*A*	*C*	*0.195*	*0.69*	*0.02064*	*0.73*	*0.06131*	*0.00308*	*0.02242*
*12*	*rs2268097*	*13752832*	*A*	*G*	*0.213*	*0.63*	*0.00303*	*0.68*	*0.0202*	*0.00018*	*0.005*
*12*	*rs2300238*	*13813330*	*A*	*G*	*0.205*	*0.63*	*0.00376*	*0.7*	*0.02959*	*0.00033*	*0.00601*
*12*	*rs980365*	*13820027*	*A*	*G*	*0.21*	*0.67*	*0.00995*	*0.81*	*0.1747*	*0.0054*	*0.02242*
*12*	*rs2268102*	*13822239*	*A*	*G*	*0.2*	*0.67*	*0.01141*	*0.81*	*0.1907*	*0.00664*	*0.02563*
*12*	*rs2284406*	*13825416*	*A*	*G*	*0.339*	*0.67*	*0.0016*	*0.97*	*0.7873*	*0.01539*	*0.04375*
*12*	*rs1008619*	*13826407*	*G*	*A*	*0.21*	*0.65*	*0.00644*	*0.82*	*0.2019*	*0.00467*	*0.02242*
*12*	*rs918065*	*13842709*	*A*	*G*	*0.192*	*0.66*	*0.01041*	*0.8*	*0.1617*	*0.00509*	*0.02242*
12	rs10845827	13859064	G	A	0.207	0.78	0.1206	0.94	0.6908	0.16793	0.2748
12	rs2284411	13866172	A	G	0.207	1.22	0.1845	1.08	0.6086	0.19345	0.29847
12	rs2300257	13868507	A	G	0.191	1.29	0.09535	1.08	0.6006	0.12125	0.22579
12	rs2268120	13877888	G	A	0.222	1.17	0.2626	1.14	0.3587	0.14954	0.25453
*12*	*rs2216128*	*13883014*	*G*	*A*	*0.175*	*0.84*	*0.2684*	*0.61*	*0.00782*	*0.00773*	*0.0261*
*12*	*rs2192973*	*13896555*	*A*	*G*	*0.162*	*0.8*	*0.1665*	*0.59*	*0.00624*	*0.00359*	*0.02242*
12	rs11055608	13913426	C	A	0.237	1.1	0.4838	1.16	0.2808	0.20848	0.31272
12	rs7301500	13941779	G	A	0.36	0.96	0.7464	0.87	0.2709	0.31384	0.41335
12	rs2284418	13943628	G	A	0.142	1.11	0.5408	1.09	0.6076	0.42628	0.50042
12	rs7974275	13950577	C	A	0.285	1.1	0.4786	1.19	0.1858	0.15083	0.25453
*12*	*rs2300266*	*13951767*	*C*	*A*	*0.15*	*0.71*	*0.05551*	*0.71*	*0.07075*	*0.00849*	*0.02625*
*12*	*rs11055625*	*13952894*	*G*	*A*	*0.15*	*0.72*	*0.05737*	*0.71*	*0.07075*	*0.00875*	*0.02625*
12	rs220573	13956734	G	A	0.452	0.95	0.6374	0.95	0.6761	0.52954	0.59573
12	rs220575	13957286	A	G	0.461	0.97	0.8137	0.97	0.7735	0.71128	0.74921
12	rs220583	13960743	A	G	0.13	1.06	0.7389	1.32	0.0876	0.14886	0.25453
12	rs220597	13968186	A	G	0.13	1.06	0.6451	1.15	0.2899	0.28281	0.39158
12	rs220599	13975298	G	A	0.13	0.93	0.5557	0.92	0.4867	0.36362	0.43634
*12*	*rs2160732*	*13981326*	*C*	*A*	*0.169*	*0.7*	*0.02686*	*0.72*	*0.06544*	*0.00413*	*0.02242*
12	rs2160734	13984349	A	G	0.3	1.21	0.1433	1.11	0.4313	0.11152	0.21507
12	rs2284424	13988870	A	G	0.275	1.21	0.1469	1.14	0.3116	0.08165	0.16957
12	rs2284425	13989019	A	C	0.313	1.3	0.04584	1.11	0.4003	0.04478	0.10513
12	rs2300273	13990434	G	A	0.354	1.31	0.0329	1.07	0.6007	0.0603	0.13308
*12*	*rs1861787*	*14000568*	*A*	*C*	*0.146*	*0.68*	*0.0247*	*0.72*	*0.08146*	*0.0048*	*0.02242*
*12*	*rs2284428*	*14009914*	*G*	*A*	*0.158*	*0.68*	*0.02298*	*0.71*	*0.06299*	*0.00347*	*0.02242*
12	rs10845852	14027137	A	C	0.157	0.82	0.2632	0.72	0.07171	0.03896	0.09563
12	rs10845853	14035011	A	G	0.368	1.08	0.5351	1.12	0.3528	0.27325	0.38831
12	rs10492141	14045250	G	A	0.193	0.72	0.03781	0.87	0.3709	0.03561	0.09158
12	rs10160840	14058573	A	G	0.193	0.73	0.04359	0.91	0.5318	0.06161	0.13308
12	rs918168	14078634	A	G	0.242	0.89	0.4054	0.69	0.01759	0.02338	0.06314
12	rs219876	14081623	A	G	0.102	1.08	0.6976	1.03	0.8639	0.69213	0.7475
12	rs1421108	14131558	G	A	0.398	1.04	0.7694	1	0.9837	0.82451	0.82452
12	rs10845868	14154639	A	C	0.408	1.03	0.8137	1.02	0.8471	0.76191	0.77628
12	rs10772722	14161665	C	A	0.409	1.03	0.8137	1.03	0.7883	0.72146	0.74921

Note: MAF represents minor allele frequency in the Chinese population; P.cc represents P values of the case-control cohort; P.trios represents P values of the trios cohort; P.comb represents P values after meta-analysis; P.adj represents adjusted P values using FDR.

**Table 2:** Results of haplotype analysis of the LD block identified by single point association analysis

HAPLOTYPE	FREQ.tios	FREQ.cc	F_A.cc	F_U.cc	T.trios	U.trios	CHISQ.cc	CHISQ.trios	P.cc	P.trios	P.comb
G	0.824	0.813	0.8494	0.8077	91	49	3.106	12.60	0.07802	3.86E-04	1.73E-04
GC	0.820	0.810	0.8567	0.8074	92	49	4.304	13.11	0.03802	2.93E-04	5.64E-05
GCG	0.806	0.810	0.8576	0.8074	107.1	58.93	4.496	13.98	0.03397	1.84E-04	3.42E-05
GCGC	0.800	0.800	0.8537	0.8038	109.1	59.93	4.365	14.31	0.03667	1.55E-04	3.29E-05
GCGCG	0.776	0.787	0.856	0.7978	119.1	63.93	5.773	16.64	0.01628	4.52E-05	4.58E-06
GCGCGG	0.772	0.782	0.8556	0.7995	122.1	61.93	5.377	19.68	0.02040	9.16E-06	1.78E-06
GCGCGGG	0.758	0.761	0.8288	0.7893	126.1	65.95	2.533	18.83	0.11150	1.43E-05	2.74E-05
GCGCGGGG	0.758	0.761	0.8372	0.7904	126.1	65.95	3.546	18.83	0.05968	1.43E-05	1.08E-05
GCGCGGGGG	0.637	0.627	0.6764	0.6541	167.1	98.39	0.573	17.77	0.44920	2.49E-05	4.38E-04
GCGCGGGGGA	0.636	0.625	0.6719	0.6537	167.1	97.38	0.384	18.36	0.53530	1.82E-05	5.23E-04
GCGCGGGGGAG	0.636	0.625	0.672	0.6565	167.1	97.39	0.278	18.36	0.59810	1.83E-05	6.67E-04

Note: Haplotype GCGCGGGGGAG involved SNPs are rs7970177|rs1805474|rs888150|rs1805510|rs2268097|rs2300238|rs980365|rs2268102|rs2284406|rs1008619|rs918065. Haplotype association analysis was performed using PLINK with a sliding window. P.cc represents P values of the case-control cohort; P.trios represents P values of the trios cohort; P.comb represents P values after meta-analysis.

**Table 3:** Common coding variants identified by Sanger sequencing and association results under an additive model

Variants	ExonicFunc	MAF_ESP6500	MAF_1000G	MAF_275case	dbSNP138	OR	P	P.adj
c.C2664T:p.T888T	synonymous	0.216	0.484	0.496	rs1806201	1.05	0.6849	0.6849
c.T4197C:p.H1399H	synonymous	0.168	0.203	0.131	rs1805247	0.59	***0.0015***	***0.0061***
c.C1806T:p.I602I	synonymous	0.039	0.187	0.123	rs1805522	0.62	***0.0042***	***0.0083***
c.C4218T:p.F1406F	synonymous	0.027	0.077	0.092	rs1805246	1.23	0.3570	0.4760

Note: MAF_1000G only included Chinese samples (CHB and CHD). P.adj represents adjusted P values using FDR.

**Table 4:** Rare coding variants identified by Sanger sequencing and association results

AAChange	ExonicFunc	num_ESP6500	num_1000G	num_275case	dbSNP138	burden	case-only
c.C2793T:p.V931V	synonymous	0	0	1	novel	*P = 0.42*	*P = 0.47*
c.C2877T:p.F959F	synonymous	0	0	3	novel		
c.A3429G:p.S1143S	synonymous	0	0	6	novel		
c.C3564G:p.G1188G	synonymous	0	0	3	novel		
c.C3683T:p.T1228M	missense	0	0	1	rs75670883		
c.A4015G:p.M1339V	missense	0	0	1	novel		
c.C3818A:p.T1273K	missense	0	0	1	novel		
